# An Epidemiologic Analysis of Co-Occurring Alcohol and Tobacco Use and Disorders: Findings From the National Epidemiologic Survey on Alcohol and Related Conditions

**Published:** 2006

**Authors:** Daniel E. Falk, Hsiao-ye Yi, Susanne Hiller-Sturmhöfel

**Affiliations:** Daniel E. Falk, Ph.D., is a research analyst and Hsiao-ye Yi, Ph.D., is a senior research analyst with the Alcohol Epidemiologic Data System of the National Institute on Alcohol Abuse and Alcoholism (NIAAA), which is operated by CSR, Incorporated, Arlington, Virginia. Susanne Hiller-Sturmhöfel, Ph.D., is senior science editor for Alcohol Research & Health

**Keywords:** Age differences, epidemiology, alcohol and other drug use disorders (AODD), racial differences, tobacco, nicotine, comorbidity, ethnic differences, gender differences

## Abstract

The 2001–2002 National Epidemiologic Survey on Alcohol and Related Conditions (NESARC) sought to determine the prevalence of drinking, smoking, and associated disorders in the general population. This survey, which includes a large representative sample of the adult population of the United States, found that drinking rates were highest among young adults and declined with increasing age. Rates of smoking and co-use of alcohol and tobacco were highest among the youngest respondents and declined thereafter. Similar patterns existed for the presence of alcohol use disorders (AUDs), nicotine dependence, and comorbidity between AUDs and nicotine dependence. Among ethnic/racial groups evaluated, Whites were most likely to drink and Native Americans/Alaskan Natives were most likely to smoke and to have an AUD, nicotine dependence, or comorbid AUD and nicotine dependence. Finally, the rates of tobacco use, daily tobacco use, and nicotine dependence increased with increasing levels of alcohol consumption and the presence of an AUD. These findings have important implications for the development of prevention and intervention approaches.

Numerous studies have demonstrated that excessive alcohol consumption and tobacco use are independently associated with a plethora of health-related, social, and economic adverse consequences for the consumers of these legal substances as well as for society at large (Centers for Disease Control and Prevention [CDC] 2004*a*Centers for Disease Control and Prevention [CDC] 2005*a*). Some of these detrimental effects can be exacerbated in people who use and abuse both substances. For example, the risk of certain cancers is greater for people who drink and smoke than for people who use only one of these substances (*a*National Institute on Alcohol Abuse and Alcoholism [NIAAA] 1998*a*). To prevent the harmful interaction of alcohol and tobacco use and ameliorate its impact on the individual and society, it is important to understand the mechanisms contributing to the use and abuse of both substances. Many studies into these mechanisms have identified brain molecules, such as neurotransmitters and nicotinic receptors, that interact with alcohol and/or nicotine and which mediate the effects of these substances on the brain. Similar analyses also have identified mechanisms that mediate sensitivity to alcohol and/or nicotine, as well as the rewarding properties of the two drugs. (For more information on these mechanisms, see the articles by Funk and colleagues, Davis and de Fiebre, and Grucza and Bierut in this issue.)

Another large area of research is concerned with how the use of or dependence on one of these substances affects the treatment outcome for dependence on the other substance. For example, studies have begun to address the following questions: Are alcohol-dependent patients who are also nicotine dependent less likely to have successful alcoholism treatment outcomes than those patients who are not nicotine dependent? Should both dependencies be treated at the same time or consecutively? And are special treatment approaches needed for patients who are dependent on both rather than just one substance? This research already has yielded valuable insights. (For more information, see the articles by Foulds and colleagues, Gulliver and colleagues, Ziedonis and colleagues, and Kodl and colleagues in this issue.)

Although all of these avenues of research are of the utmost importance, it is equally critical to obtain a solid understanding of the scope of the problem. Thus, it is important to know the rates of alcohol and tobacco use and co-use in the general population and whether certain demographic subgroups are particularly at risk of using both alcohol and tobacco or being dependent on both substances. Studies of this nature are rare in the literature. In a previous issue of *Alcohol Research & Health*, Anthony and Echeagaray-Wagner (2000) presented an epidemiologic analysis of alcohol and tobacco use and co-use using national data collected before 1997. This analysis found that the prevalence of co-occurring alcohol and tobacco use and disorders was lowest among young adolescents, peaked among young adults, and declined thereafter. The peak rate of alcohol and tobacco co-use among young adults ranged from 35 to 45 percent, and the peak rate of co-dependence was approximately 10 percent.

In addition to determining the prevalence of co-occurring alcohol and tobacco use and disorders in the general population and for certain demographic subgroups, it is important to understand how rates of tobacco use and nicotine dependence vary among people with different characteristics of alcohol consumption. Over the last 20 years or more, accumulating evidence has indeed pointed to a dose-response relationship between alcohol and tobacco use:

- Dawson (2000) reported that people who abuse or are dependent on alcohol are more likely to use tobacco and are heavier users than people who abstain from alcohol.
- DiFranza and Guerrera (1990) found that 83 percent of alcoholics were smokers, compared with 34 percent of nonalcoholics.
- Bobo and Husten (2000) showed that 37 percent of adults who were classified as current drinkers were also current smokers, compared with 13 percent of abstainers.
- Other analyses indicated that smoking rates and the number of cigarettes smoked increase with increasing alcohol consumption (e.g., Friedman et al. 1991).

Although data such as these strongly point to an association between drinking and smoking, the existing studies often have characteristics that, while making them useful for addressing specific research questions, limit their generalizability to the general population. These characteristics include the use of treatment samples, local community samples, or special subpopulation samples rather than nationally representative samples. Moreover, most comorbidity studies focused on smoking or cigarette smoking as the measure of tobacco use (e.g., Dawson 2000; Anthony and Echeagaray-Wagner 2000; Friedman et al. 1991), ignoring other modalities of tobacco use (e.g., the use of snuff and chewing tobacco). In addition, many of the existing studies were based on surveys conducted in the 1980s and have used diagnostic criteria that are no longer current. Particularly with the prevalence of tobacco use declining dramatically in the United States in recent decades (Marks et al. 1997; CDC 2005*b*), epidemiological studies based on current national data are needed to provide updated estimates of alcohol and tobacco co-use in the general population.

This article addresses these issues by updating the findings presented by Anthony and Echeagaray-Wagner (2000) using data from the 2001–2002 National Epidemiologic Survey on Alcohol and Related Conditions (NESARC). The main objective is to present data on the prevalence and pattern of alcohol and tobacco use and co-use in the United States. This analysis is based on a comprehensive definition of tobacco use including all tobacco modalities, with all prevalence estimates presented by gender, race/ethnicity, and age. In addition, the present study expands upon the work of Anthony and Echeagaray-Wagner (2000) by analyzing how tobacco use and nicotine dependence vary with levels of alcohol consumption and types of alcohol use disorders (AUDs).

## Methods

### Data

The present study is based on data from the first wave of the NIAAA-sponsored NESARC, which was conducted in 2001–2002. NESARC is among the largest nationally representative comorbidity surveys ever conducted and includes extensive questions on alcohol and tobacco (as well as other substance) use and related disorders. The NESARC sample includes 43,093 respondents ages 18 and older, representing the civilian, noninstitutionalized adult population of the United States, including residents of all 50 States and the District of Columbia. Military personnel living off base and persons living in noninstitutionalized group-quarter housing, such as boarding houses, shelters, and dormitories, also were included (Grant et al. 2003). The sampling frames for housing units and group-quarter units were derived from the Census 2000/2001 Supplementary Survey and the Census 2000 Group Quarters Inventory, respectively. NESARC oversampled Blacks, Hispanics, and young adults (ages 18–24) to allow for more reliable estimates of these groups. Data were collected via face-to-face, computer-assisted interviews in household settings. From each household, one adult was selected for interview. The overall response rate for NESARC was 81 percent.

### Measures

#### Alcohol Measures

All alcohol measures reflect consumption and diagnostic status during the past year (i.e., the 12 months preceding the NESARC interview). Although NESARC collected alcohol data separately for each of four beverage types (i.e., wine, beer, coolers, and distilled spirits), the results presented here are the aggregate across beverage types. The various alcohol measures are defined as follows:

- *Lifetime abstainer—*Never had one or more drinks of alcohol during the course of a lifetime, excluding small tastes or sips.
- *Former drinker—*Had at least one drink prior to the past year but no drinks during the past year.
- *Current drinker (or alcohol use)—* Had at least one drink of alcohol during the past year. Based on past-year consumption, current drinkers are divided into three drinking levels:
  *– Light drinker—*Had three or fewer drinks per week.
  *– Moderate drinker—*Had 4–14 drinks per week for men and 4–7 drinks per week for women.
  *– Heavy drinker—*Had more than 14 drinks per week for men and more than 7 drinks per week for women.
- *AUD*—Met the criteria articulated in the *Diagnostic and Statistical Manual of Mental Disorders, Fourth Edition* (DSM–IV) (American Psychiatric Association 1994) for alcohol abuse, alcohol dependence, or both during the past year. AUDs were diagnosed using NIAAA’s Alcohol Use Disorder and Associated Disabilities Interview Schedule–DSM–IV Version (AUDADIS–IV) (Grant et al. 2001), an interview instrument based on the DSM–IV criteria. Diagnoses of AUDs in NESARC are defined as follows:
  *– Alcohol abuse (AA)—*Met at least one of the following four criteria for DSM–IV alcohol abuse (and did not meet criteria for alcohol dependence): continued use despite social or interpersonal consequences, hazardous use, alcohol-related legal consequences, or neglect of role responsibilities in favor of drinking.
  *– Alcohol dependence (with or without alcohol abuse) (AD)—*Met at least three of the following seven criteria for DSM–IV alcohol dependence in the same 12-month period: tolerance, withdrawal syndrome or drinking to relieve/avoid withdrawal, impaired control over drinking, persistent desire or unsuccessful attempts to cut down or stop drinking, much time spent drinking, reducing/giving up important activities in favor of drinking, and continued drinking despite physical or psychological problems caused by drinking.

#### Tobacco Measures

Like the alcohol measures above, the tobacco measures employed in the present study also reflect past-year use and diagnostic status. NESARC collected data separately for each of four tobacco modalities (i.e., cigarettes, cigars, pipes, and snuff and chewing tobacco); however, the analyses presented here are an aggregate across all modalities. The various tobacco measures are defined as follows:

- *Tobacco use*—Had smoked at least 100 cigarettes or 50 cigars, had smoked a pipe at least 50 times, or had used snuff or chewing tobacco at least 20 times during the course of a lifetime and had used any tobacco modality at least once during the past year.
- *Daily tobacco use*—Had used at least one tobacco modality at least once every day during the past year.
- *Nicotine dependence*^1^—Met at least three of the seven DSM–IV criteria for nicotine dependence in the past year. Similar to alcohol dependence, nicotine dependence was diagnosed using the AUDADIS–IV. The diagnostic criteria of nicotine dependence include tolerance, withdrawal syndrome or using tobacco to relieve/avoid withdrawal symptoms, using tobacco more than intended, persistent desire or unsuccessful attempts to cut down or stop tobacco use, great deal of time spent using tobacco, giving up activities in favor of tobacco use, and continued using tobacco despite physical or psychological problems caused by its use. Many of the specific symptom items used to operationalize nicotine dependence criteria are different from those for alcohol dependence (Grant et al. 2004).

#### Co-Occurrence of Alcohol and Tobacco Measures

Based on past-year alcohol and tobacco use and disorder status, two measures are defined:

- *Co-use—*Had used both alcohol and tobacco within the past year.
- *Comorbidity—*Had both an AUD and nicotine dependence in the past year.

### Data Analysis

Data presented in this article are descriptive in nature. All prevalence estimates of alcohol and tobacco use and disorders are presented as percentages of the total adult population in the United States or of a subpopulation group. All analyses are carried out for men and women separately. Rates of alcohol and tobacco use, co-use, disorders, and comorbidity are estimated for four age-groups (ages 18–24, 25–44, 45–64, and 65 and older) and five mutually exclusive racial/ethnic groups (i.e., White, Black, American Indian/Alaskan Native, Asian/Native Hawaiian/Pacific Islander, and Hispanic). Furthermore, to evaluate the relationship between drinking characteristics and tobacco use, the prevalence of tobacco use, daily tobacco use, and nicotine dependence is presented by drinking categories (i.e., lifetime abstainer, former drinker, and current drinker). Current drinkers are further divided into five mutually exclusive subgroups (i.e., light drinking, moderate drinking, heavy drinking, alcohol abuse, and alcohol dependence). All estimates were weighted by sampling weights to represent the entire adult population of the United States. Standard errors for all estimates were generated using the software package SUDAAN, which takes into account the effect of the NESARC complex sampling design (Research Triangle Institute 2004).

## Results

### Prevalence of Alcohol and Tobacco Use and Co-Use

Table 1 presents the national prevalence estimates of alcohol and tobacco use as well as their co-use, by age and race/ethnicity, for men and women separately. According to these data, approximately two-thirds of adults in the United States used alcohol and more than one-quarter used tobacco during the past year. Furthermore, 21.7 percent of adults in the United States used both alcohol and tobacco, representing approximately 46.2 million adults. Men and women had similar rates of using alcohol only (44.4 and 43.2 percent, respectively) and using tobacco only (6.4 and 5.6 percent, respectively) (Figure 1a). However, men were much more likely than women to use both alcohol and tobacco (27.5 vs. 16.4 percent, respectively), whereas women were more likely than men to abstain from both substances (34.9 vs. 21.8 percent, respectively).

#### Prevalence by Age-Group

The prevalence of alcohol use by age-group showed a somewhat curvilinear pattern for both men and women (Figure 1b). Alcohol use was common among people ages 18–24 (74.8 and 66.8 percent for men and women, respectively), peaked at ages 25–44 (77.7 and 68.3 percent, respectively), began to decline among people ages 45–64 (70.4 and 58.5 percent, respectively), and was lowest among people ages 65 and older (55.4 and 37.6 percent, respectively).

In contrast, tobacco use declined monotonically for both genders, with the highest prevalence among the youngest group (38.6 and 28.4 percent for men and women, respectively) and the lowest prevalence among the oldest group (18.2 and 10.4 percent, respectively). Similarly, the prevalence of co-use of alcohol and tobacco declined steadily with age, from the highest rate among people ages 18–24 (34.8 and 25.5 percent for men and women, respectively) to the lowest rate among people ages 65 and older (11.3 and 4.6 percent, respectively).

#### Prevalence by Race/Ethnicity

The prevalence of alcohol use varied among racial/ethnic groups for both men and women. For both genders, Whites displayed the highest rates (74.3 and 65.1 percent for men and women, respectively) and Asians/Native Hawaiians/Pacific Islanders displayed the lowest rates (61.5 and 36.1 percent, respectively) (Figure 1c). The prevalence of tobacco use for both genders was highest among American Indians/Alaskan Natives (49.6 and 34.1 percent for men and women, respectively) and lowest among Asians/Native Hawaiians/Pacific Islanders (22.2 and 8.9 percent, respectively). Parallel to this pattern, the prevalence of using both alcohol and tobacco was highest among American Indians/Alaskan Natives (34.0 percent among men and 22.8 percent among women) and Asians/Native Hawaiians/Pacific Islanders had the lowest prevalence (18.1 and 6.6 percent, respectively). Whites, Blacks, and Hispanics had intermediate levels of co-use.

### Prevalence of AUDs, Nicotine Dependence, and Comorbidity

NESARC provides national prevalence estimates of AUDs, nicotine dependence, and their comorbidity, by age and race/ethnicity for men and women separately. According to these data, 8.4 percent of adults in the United States had an AUD and 12.8 percent had nicotine dependence during the past year (Table 2, Figure 2a). Moreover, 2.9 percent of all adults had a comorbid AUD and nicotine dependence, representing approximately 6.2 million adults. Additional analysis found that the prevalence of comorbid alcohol and nicotine dependence was slightly higher than the prevalence of comorbid alcohol abuse and nicotine dependence (1.7 vs. 1.2 percent, respectively) (Figure 2a). Although men and women had similar rates of nicotine dependence only (10.0 and 9.7 percent, respectively), men were more likely than women to have an AUD only (8.2 vs. 3.1 percent, respectively) and comorbid disorders (4.1 vs. 1.8 percent, respectively).

#### Prevalence by Age-Group

For both genders, the prevalence of AUDs, nicotine dependence, and comorbid disorders decreased with age (Figure 2b). The prevalence of AUDs was highest among the youngest men and women (25.1 and 11.7 percent, respectively) and lowest among the oldest men and women (2.8 and 0.5 percent, respectively). Similarly, the prevalence of nicotine dependence was highest among the youngest men and women (19.1 and 15.2 percent, respectively) and lowest among the oldest men and women (4.6 and 3.7 percent, respectively). For each age-group, nicotine dependence generally was more prevalent than AUDs, except for men ages 18–24, for whom AUDs were more prevalent than nicotine dependence (25.1 vs. 19.1 percent, respectively).

Like the rates of AUDs and nicotine dependence, the prevalence of comorbid disorders decreased with age, with the highest prevalence found among the youngest men and women (9.9 and 4.7 percent, respectively) and the lowest prevalence found among the oldest men and women (0.4 and 0.1 percent, respectively). Finally, for all diagnoses and age-groups evaluated, prevalence of AUDs and/or nicotine dependence was higher among men than among women.

#### Prevalence by Race/Ethnicity

For AUDs, American Indians/Alaskan Natives displayed the highest prevalence (15.9 and 8.7 percent for men and women, respectively) and Asians/Native Hawaiians/Pacific Islanders had the lowest rates (6.8 and 2.5 percent, respectively) (Figure 2c). The prevalence of nicotine dependence showed greater variability among the racial/ethnic groups. For both men and women, the highest rates were found among American Indians/Alaskan Natives (26.4 and 20.2 percent, respectively). Among men, Hispanics had the lowest rate (7.0 percent), whereas among women, Asians/Native Hawaiians/Pacific Islanders had the lowest rate (4.5 percent). Across all racial/ethnic groups, the prevalence of nicotine dependence generally was greater than the prevalence of AUDs with the exception of Hispanic men, in whom the prevalence of AUDs (12.1 percent) exceeded that of nicotine dependence (7.0 percent).

For comorbid AUDs and nicotine dependence, the rank ordering pattern among the racial/ethnic groups was similar for both genders: American Indians/Alaskan Natives displayed the highest prevalence (7.0 and 4.0 percent for men and women, respectively) and Hispanics had the lowest rates (2.2 and 0.9 percent, respectively).

### Prevalence of Tobacco Use, Daily Tobacco Use, and Nicotine Dependence by Drinking Characteristics

**Figure 3:**
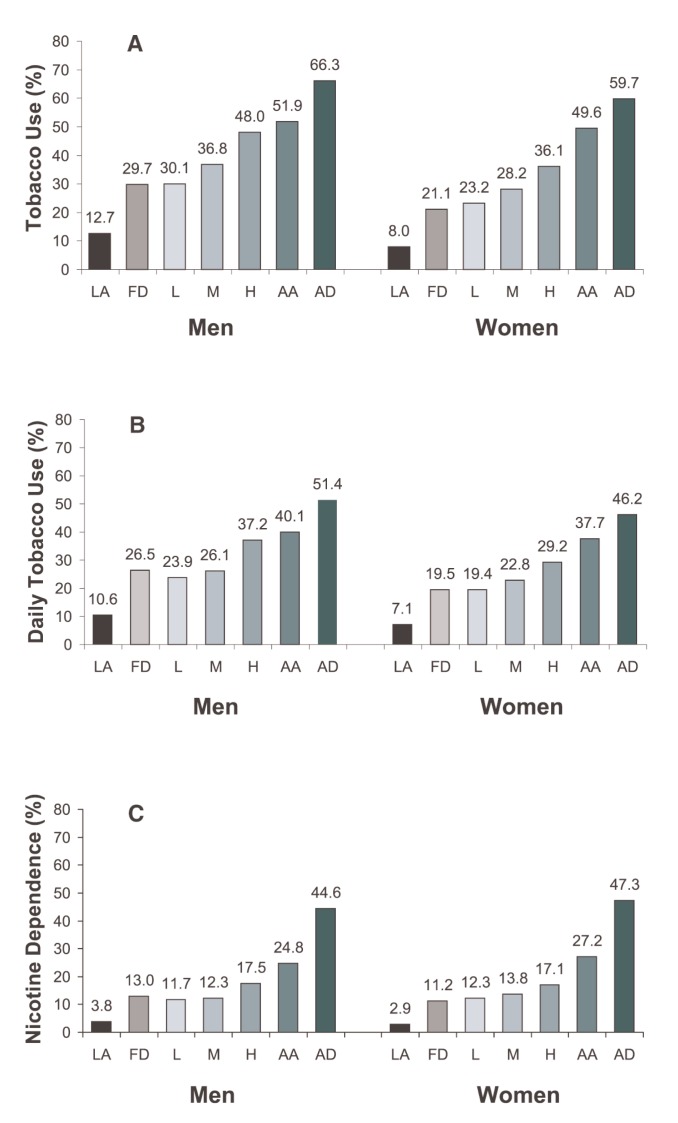
A Prevalence of past-year tobacco use by past-year drinking characteristics and gender in the United States, 2001–2002 NESARC. **B** Prevalence of past-year daily tobacco use by past-year drinking characteristics and gender in the United States, 2001–2002 NESARC. **C** Prevalence of past-year nicotine dependence by past-year drinking characteristics and gender in the United States, 2001–2002 NESARC. NOTE: Data are drawn from Table 3. LA = lifetime abstainer; FD = former drinker; L = light drinker; M = moderate drinker; H = heavy drinker; AA = alcohol abuse; AD = alcohol dependence.

NESARC data also demonstrate how tobacco use is related with the characteristics of alcohol consumption. For all three tobacco measures—that is, tobacco use, daily tobacco use, and nicotine dependence— the rates increased monotonically with increasing levels of alcohol consumption and the presence of alcohol abuse and dependence (Table 3, Figures 3A–C). For instance, among both men and women, the prevalence of tobacco use was lowest among lifetime alcohol abstainers (12.7 and 8.0 percent, respectively), increased with levels of alcohol consumption, and peaked among alcohol-dependent individuals (66.3 and 59.7 percent, respectively) (Figure 3A). Similar positive trends also were observed between drinking characteristics and daily tobacco use (Figure 3B) or nicotine dependence (Figure 3C). The association between alcohol dependence and nicotine dependence was particularly strong. Compared with the prevalence of nicotine dependence among male and female lifetime alcohol abstainers (3.8 and 2.9 percent, respectively), the prevalence of nicotine dependence among alcohol-dependent men and women was much higher (44.6 and 47.3 percent, respectively), representing an 11.7 and 16.3 times increased risk for nicotine dependence, respectively.

## Discussion and Conclusion

This study presents updated epidemiologic data on alcohol and tobacco co-use and comorbidity in the adult population of the United States. With approximately 46.2 million American adults having used both substances in the past year, the sheer extent of co-use is high. The number for comorbid disorders is considerably lower, yet still significant, with approximately 6.2 million American adults having both an AUD and nicotine dependence. Furthermore, higher rates of co-use and comorbidity were found among certain demographic subgroups. For example, similar to Anthony and Echeagaray-Wagner (2000), the current analysis revealed that the prevalence of co-use and comorbidity was higher in men than women. Moreover, the highest rates were seen in the youngest age-groups, with a steady decline observed in older age-groups. Among the racial/ethnic subpopulations, American Indians/Alaskan Natives had the highest rates of alcohol and tobacco co-use and comorbidity among both men and women. This last finding is particularly important, despite being rarely reported in the literature. Overall, the higher rates of co-use and comorbidity found in these demographic subgroups were not surprising, given reports in the literature of higher rates of alcohol use, tobacco use, AUDs, and nicotine dependence among these subpopulations (American Lung Association 2006; CDC 2005*a*; NIAAA 1998*a*,*b*). Results from the current study suggest that prevention and treatment strategies for comorbid alcohol and tobacco use disorders should place a special emphasis on men and focus efforts on other high-risk groups such as young adults and American Indians/Alaskan Natives.

In addition to quantifying the current rates of co-use and comorbidity in the United States, the present study also investigated the association between the characteristics of alcohol and tobacco use. Results showed a dose-response relationship between the two substances, with rates of tobacco use, daily tobacco use, and nicotine dependence increasing monotonically with increasing levels of alcohol consumption and the presence of AUDs. This dose-response relationship is consistent with findings noted in other epidemiological studies, although those studies mostly examined cigarette smoking rather than tobacco use in general. For example, in a national study, Dawson (2000) showed that the prevalence of past-year smoking was lowest among lifetime alcohol abstainers (23 percent) and subsequently increased to 31, 39, and 53 percent among light, moderate, and heavy drinkers, respectively. In a community sample, Friedman et al. (1991) reported a similar doseresponse gradient in both White and Black men and women, with smoking rates ranging from 23 to 26 percent among abstainers, 38 to 65 percent among moderate drinkers, and 54 to 92 percent among heavy drinkers consuming nine or more drinks per day. Similarly, Soeken and Bausell (1989) showed a relatively linear increase in smoking rates in a national sample of adults ranging from 18 percent among abstainers to 27 and 38 percent among moderate and heavy drinkers, respectively. The differences in the estimated rates of smoking among alcohol consumption groups in these studies may result from differing operational definitions of smoking and levels of alcohol consumption, the use of community versus national samples, and changing trends in surveys conducted in different years.

The present study improves on a significant portion of the existing studies by including all modalities of tobacco use rather than focusing only on cigarette smoking. The rationale for including all modalities of tobacco use is that all modalities have been shown to have detrimental health consequences when used in excess (CDC 2004, 2005*c*). For those wishing to draw comparisons to studies involving only cigarette smoking, it is noteworthy that separate analyses revealed that cigarette smoking accounted for approximately 88 percent of all tobacco use. Thus, the prevalence estimates of alcohol and tobacco use and co-use presented in this study provide health care policymakers and treatment planners with a comprehensive assessment of the state of the use and dual-use problem in America. Nonetheless, the current study is descriptive in nature. Given the high rates of co-use and comorbidity in this country, continued research into the mechanisms underlying co-use and comorbidity certainly are warranted.

## Figures and Tables

**Figure 1a:**
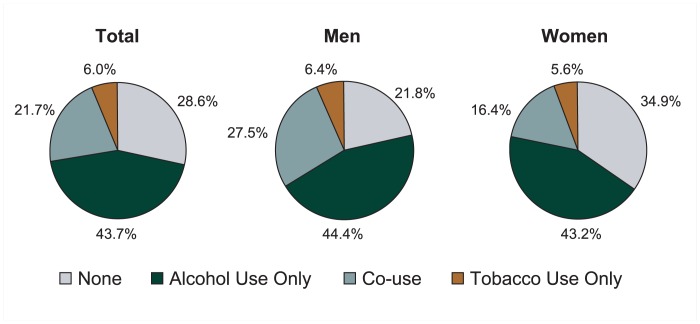
Prevalence of past-year alcohol and tobacco use and co-use in the United States, 2001–2002 NESARC. NOTE: Data are drawn from Table 1. The prevalence of alcohol or tobacco use only is derived as follows: % alcohol use only = % alcohol use – % co-use; % tobacco use only = % tobacco use – % co-use.

**Figure 1b:**
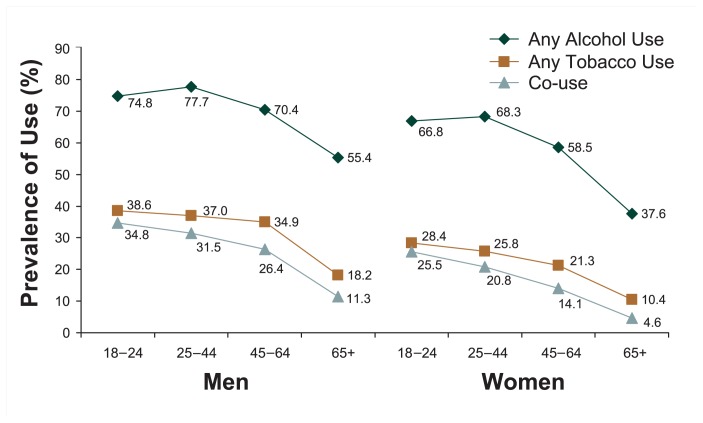
Prevalence (%) of past-year alcohol use, tobacco use, and co-use by age and gender in the United States, 2001–2002 NESARC.

**Figure 1c:**
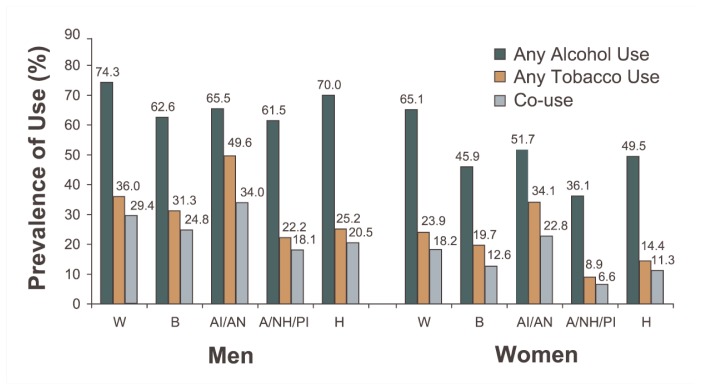
Prevalence of past-year alcohol use, tobacco use, and co-use by race/ethnicity and gender in the United States, 2001–2002 NESARC. NOTE: Data are drawn from Table 1. W = White; B = Black; AI/AN = American Indian/Alaskan Native; A/NH/PI = Asian/Native Hawaiian/Pacific Islander; H = Hispanic.

**Figure 2a:**
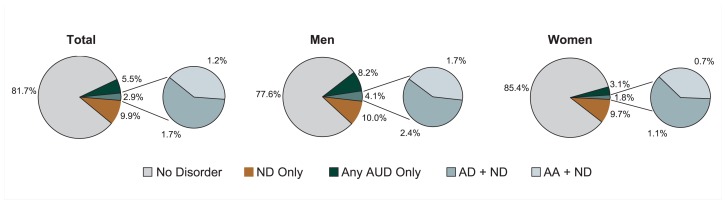
Prevalence of past-year alcohol use disorder, nicotine dependence, and comorbid disorders in the United States, 2001–2002 NESARC. NOTE 1: Data are drawn from Table 2. ND = nicotine dependence; AUD = alcohol use disorder; AD = alcohol dependence; AA = alcohol abuse. NOTE 2: The prevalence of AUDs or ND only is derived as follows: % AUD only = % AUD – % comorbid; % ND only = % ND – % comorbid.

**Figure 2b:**
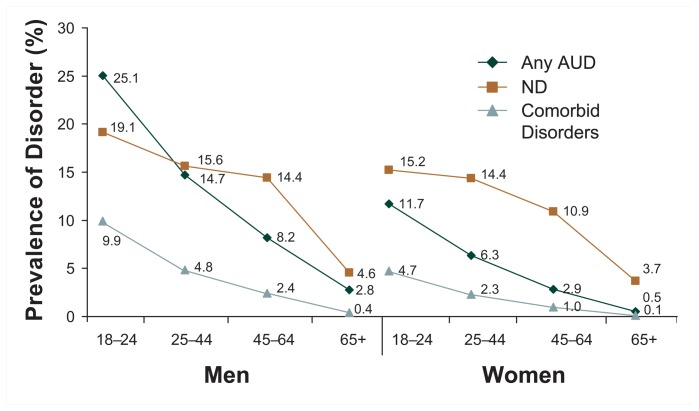
Prevalence (%) of any past-year alcohol use disorder, nicotine dependence, and comorbid disorders by age and gender in the United States, 2001–2002 NESARC. NOTE 1: Data are drawn from Table 2. AUD = alcohol use disorder; ND = nicotine dependence. NOTE 2: The prevalence of AUD or ND only is derived as follows: % AUD only = % AUD – % comorbid; % ND only = % ND – % comorbid.

**Figure 2c:**
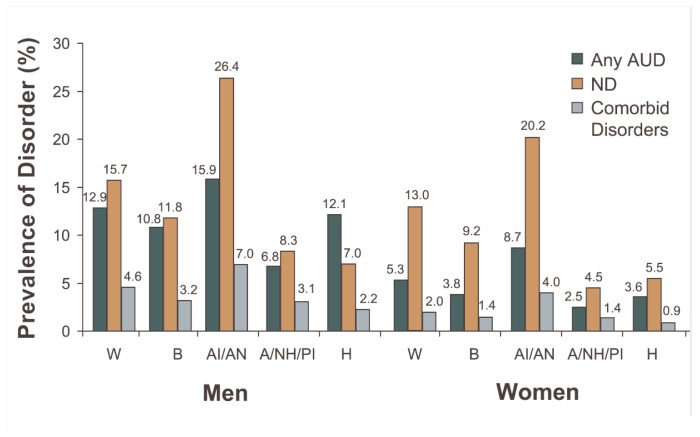
Prevalence (%) of any past-year alcohol use disorder, nicotine dependence, and comorbid disorders by race/ethnicity and gender in the United States, 2001–2002 NESARC. NOTE: Data are drawn from Table 2. W = White; B = Black; AI/AN = American Indian/Alaskan Native; A/NH/PI = Asian/Native Hawaiian/Pacific Islander; H = Hispanic; AUD = alcohol use disorder; ND = nicotine dependence.

**Table 1:** Prevalence (%) of Past-Year Alcohol and Tobacco Use and Co-Use in the United States, By Gender, Age, and Race/Ethnicity, 2001–2002 NESARC

	Alcohol/Tobacco Use							

	No Use		Any Alcohol Use		Any Tobacco Use		Co-Use	
	%	SE	%	SE	%	SE	%	SE

**Total**	28.6	0.3	65.4	0.3	27.7	0.3	21.7	0.3
**Men**	21.8	0.4	71.8	0.4	33.9	0.4	27.5	0.4
**Age**								
18–24	21.4	1.0	74.8	1.1	38.6	1.2	34.8	1.2
25–44	16.8	0.5	77.7	0.6	37.0	0.7	31.5	0.7
45–64	21.1	0.7	70.4	0.8	34.9	0.8	26.4	0.7
65+	37.8	1.0	55.4	1.0	18.2	0.8	11.3	0.6
**Race/Ethnicity**								
White	19.2	0.4	74.3	0.5	36.0	0.5	29.4	0.5
Black	30.9	1.0	62.6	1.1	31.3	1.1	24.8	1.0
American Indian/Alaskan Native	18.9	2.6	65.5	3.5	49.6	3.1	34.0	3.2
Asian/Native Hawaiian/Pacific Islander	34.4	2.3	61.5	2.4	22.2	2.1	18.1	1.9
Hispanic	25.4	1.1	70.0	1.2	25.2	1.0	20.5	0.9
**Women**	34.9	0.4	59.6	0.4	22.0	0.3	16.4	0.3
**Age**								
18–24	30.3	1.1	66.8	1.1	28.4	1.1	25.5	1.0
25–44	26.7	0.6	68.3	0.6	25.8	0.6	20.8	0.5
45–64	34.3	0.7	58.5	0.7	21.3	0.6	14.1	0.5
65+	56.5	0.8	37.6	0.8	10.4	0.5	4.6	0.4
**Race/Ethnicity**								
White	29.2	0.5	65.1	0.5	23.9	0.4	18.2	0.4
Black	47.0	0.9	45.9	0.9	19.7	0.7	12.6	0.6
American Indian/Alaskan Native	37.0	3.0	51.7	3.4	34.1	2.9	22.8	2.7
Asian/Native Hawaiian/Pacific Islander	61.6	2.1	36.1	2.1	8.9	1.1	6.6	1.0
Hispanic	47.4	1.1	49.5	1.1	14.4	0.7	11.3	0.6

SE = standard error of the estimated percentage.

**Table 2:** Prevalence (%) of Past-Year Alcohol Use Disorders, Nicotine Dependence, and Comorbid Disorders in the United States, by Gender, Age, and Race/Ethnicity, 2001–2002 NESARC

	No Disorder		AUD						ND		Comorbidity					
			
			AA		AD		Any AUD				AA w/ND		AD w/ND		Any AUD w/ND	

	%	SE	%	SE	%	SE	%	SE	%	SE	%	SE	%	SE	%	SE

**Total**	81.7	0.2	4.7	0.1	3.8	0.1	8.5	0.2	12.8	0.2	1.2	0.1	1.7	0.1	2.9	0.1
**Men**	77.6	0.4	6.9	0.2	5.4	0.2	12.4	0.3	14.2	0.3	1.7	0.1	2.4	0.1	4.1	0.2
**Age**																
18–24	65.7	1.2	8.7	0.7	16.4	0.9	25.1	1.1	19.1	1.0	2.2	0.4	7.7	0.7	9.9	0.8
25–44	74.5	0.6	9.1	0.4	5.6	0.3	14.7	0.5	15.6	0.5	2.4	0.3	2.4	0.2	4.8	0.3
45–64	79.9	0.7	5.5	0.4	2.7	0.2	8.2	0.4	14.4	0.6	1.2	0.2	1.2	0.2	2.4	0.2
65+	93.1	0.5	2.4	0.3	0.4	0.1	2.8	0.3	4.6	0.4	0.3*	0.1	0.1*	0.1	0.4	0.1
**Race/Ethnicity**																
White	76.0	1.2	7.5	0.3	5.4	0.9	12.9	0.4	15.7	0.4	2.0	0.2	2.6	0.2	4.6	0.3
Black	80.6	0.6	5.7	0.5	5.1	0.3	10.8	0.7	11.8	0.7	1.4	0.3	1.8	0.3	3.2	0.4
American Indian/Alaskan Native	64.7	0.7	7.5	1.6	8.4	0.2	15.9	2.4	26.4	3.2	2.0*	1.0	5.0	1.5	7.0	1.8
Asian/Native Hawaiian/Pacific Islander	88.0	0.5	3.2	0.8	3.6	0.1	6.8	1.2	8.3	1.3	1.0*	0.4	2.1*	0.7	3.1	0.8
Hispanic	83.1	0.9	6.2	0.5	5.9	0.6	12.1	0.8	7.0	0.6	0.7	0.2	1.5	0.3	2.2	0.4
**Women**	85.4	0.3	2.6	0.1	2.3	0.1	4.9	0.2	11.5	0.3	0.7	0.1	1.1	0.1	1.8	0.1
**Age**																
18–24	77.8	1.0	4.8	0.5	6.9	0.6	11.7	0.8	15.2	0.9	1.6	0.3	3.1	0.4	4.7	0.5
25–44	81.6	0.5	3.5	0.2	2.8	0.2	6.3	0.3	14.4	0.5	0.9	0.1	1.4	0.2	2.3	0.2
45–64	87.2	0.5	1.7	0.2	1.2	0.2	2.9	0.3	10.9	0.5	0.5	0.1	0.5	0.1	1.0	0.2
65+	95.9	0.3	0.4	0.1	0.1*	0.1	0.5	0.1	3.7	0.3	0.0*	0.0	0.1*	0.1	0.1*	0.1
**Race/Ethnicity**																
White	83.7	0.4	2.9	0.2	2.4	0.2	5.3	0.2	13.0	0.3	0.8	0.1	1.1	0.1	2.0	0.1
Black	88.4	0.6	1.4	0.2	2.4	0.3	3.8	0.4	9.2	0.5	0.2*	0.1	1.2	0.2	1.4	0.2
American Indian/Alaskan Native	75.1	2.5	4.2*	1.3	4.5	1.2	8.7	1.7	20.2	2.4	1.1*	0.6	2.9*	1.0	4.0	1.1
Asian/Native Hawaiian/Pacific Islander	94.4	0.9	1.1*	0.4	1.3*	0.5	2.5	0.6	4.5	0.8	0.8*	0.4	0.6*	0.3	1.4*	0.5
Hispanic	91.8	0.5	1.7	0.3	1.9	0.3	3.6	0.4	5.5	0.5	0.3*	0.1	0.6	0.2	0.9	0.2

NOTE: AA = alcohol abuse ; AD = alcohol dependence; AUD = alcohol use disorder; ND = nicotine dependence; SE = standard error of the estimated percentage.

**Table 3:** Prevalence (%) of Past-Year Tobacco Use, Daily Tobacco Use, and Nicotine Dependence by Drinking Characteristics and Alcohol Use Disorders in the United States, 2001–2002 NESARC

	Any Tobacco Use		Daily Tobacco Use		Nicotine Dependence	

	%	SE	%	SE	%	SE

**Total**	27.7	0.3	22.4	0.3	12.8	0.2
**Male**	33.9	0.4	26.7	0.4	14.2	0.3
Lifetime Abstainer	12.7	0.9	10.6	0.9	3.8	0.5
Former Drinker	29.7	1.0	26.5	1.0	13.0	0.8
Current Drinker	38.2	0.5	29.3	0.5	16.1	0.4
Light	30.1	0.7	23.9	0.7	11.7	0.5
Moderate	36.8	1.0	26.1	0.9	12.3	0.7
Heavy	48.0	1.7	37.2	1.6	17.5	1.3
Any AUD	58.2	1.3	45.0	1.3	33.5	1.3
Alcohol Abuse	51.9	1.7	40.1	1.7	24.8	1.6
Alcohol Dependence	66.3	1.8	51.4	1.9	44.6	1.9
**Female**	22.0	0.3	18.5	0.3	11.5	0.3
Lifetime Abstainer	8.0	0.4	7.1	0.4	2.9	0.3
Former Drinker	21.1	0.8	19.5	0.7	11.2	0.6
Current Drinker	27.5	0.5	22.6	0.4	14.8	0.4
Light	23.2	0.5	19.4	0.5	12.3	0.4
Moderate	28.2	1.4	22.8	1.3	13.8	1.1
Heavy	36.1	1.7	29.2	1.6	17.1	1.3
Any AUD	54.4	1.8	41.8	1.8	36.8	1.8
Alcohol Abuse	49.6	2.4	37.7	2.4	27.2	2.3
Alcohol Dependence	59.7	2.6	46.2	2.7	47.3	2.7

NOTE: SE = standard error of the estimated percentage; AUD = alcohol use disorder.

## Footnotes

1 Although the DSM–IV defines both alcohol abuse and alcohol dependence, it only includes a diagnosis for nicotine dependence.

## References

[b1-162-171] American Lung Association (2006). Smoking 101. Fact Sheet.

[b2-162-171] American Psychiatric Association (APA) (1994). Diagnostic and Statistical Manual of Mental Disorders, Fourth Edition (DSM–IV).

[b3-162-171] Anthony JC, Echeagaray-Wagner F (2000). Epidemiologic analysis of alcohol and tobacco use. Alcohol Research & Health.

[b4-162-171] Bobo JK, Husten C (2000). Sociocultural influences on smoking and drinking. Alcohol Research & Health.

[b5-162-171] Centers for Disease Control and Prevention (CDC) (2004). The Health Consequences of Smoking: A Report of the Surgeon General.

[b6-162-171] Centers for Disease Control and Prevention (CDC) (2005a). General Alcohol Information. Fact Sheet.

[b7-162-171] Centers for Disease Control and Prevention (CDC) (2005b). Cigarette smoking among adults: United States, 2004. Morbidity and Mortality Weekly Reports.

[b8-162-171] Centers for Disease Control and Prevention (CDC) (2005c). Smokeless Tobacco. Fact Sheet.

[b9-162-171] Dawson DA (2000). Drinking as a risk factor for sustained smoking. Drug and Alcohol Dependence.

[b10-162-171] DiFranza JR, Guerrera MP (1990). Alcoholism and smoking. Journal of Studies on Alcohol.

[b11-162-171] Friedman GD, Tekawa I, Klatsky AL, Sidney S, Armstrong MA (1991). Alcohol drinking and cigarette smoking: An exploration of the association in middle-aged men and women. Drug and Alcohol Dependence.

[b12-162-171] Grant BF, Dawson DA, Hasin DS (2001). The Alcohol Use Disorder and Associated Disabilities Interview Schedule: DSM–IV Version.

[b13-162-171] Grant BF, Moore TC, Shepard J, Kaplan K (2003). Source and Accuracy Statement for the National Epidemiologic Survey on Alcohol and Related Conditions (NESARC): Wave I.

[b14-162-171] Grant BF, Hasin DS, Chou P, Stinson FS, Dawson DA (2004). Nicotine dependence and psychiatric disorders in the United States: Results from the National Epidemiologic Survey on Alcohol and Related Conditions. Archives of General Psychiatry.

[b15-162-171] Marks JL, Hill EM, Pomerleau CS, Mudd SA, Blow FC (1997). Nicotine dependence and withdrawal in alcoholic and nonalcoholic ever-smokers. Journal of Substance Abuse Treatment.

[b16-162-171] National Institute on Alcohol Abuse and Alcoholism (NIAAA) (1998a). Alcohol and Tobacco. Alcohol Alert.

[b17-162-171] National Institute on Alcohol Abuse and Alcoholism (NIAAA) (1998b). Drinking in the United States: Main Findings from the 1992 National Longitudinal Alcohol Epidemiologic Survey (NLAES). U.S. Alcohol Epidemiologic Data Reference Manual.

[b18-162-171] Research Triangle Institute (2004). SUDAAN Language Manual, Release 9.0.

[b19-162-171] Soeken KL, Bausell RB (1989). Alcohol use and its relationship to other addictive and preventive behaviors. Addictive Behaviors.

